# Berberine Protects against Neuronal Damage via Suppression of Glia-Mediated Inflammation in Traumatic Brain Injury

**DOI:** 10.1371/journal.pone.0115694

**Published:** 2014-12-29

**Authors:** Chien-Cheng Chen, Tai-Ho Hung, Chao Yu Lee, Liang-Fei Wang, Chun-Hu Wu, Chia-Hua Ke, Szu-Fu Chen

**Affiliations:** 1 Department of Physical Medicine and Rehabilitation, Cheng Hsin General Hospital, Taipei, Taiwan, Republic of China; 2 Department of Obstetrics and Gynecology, Chang Gung Memorial Hospital at Taipei and College of Medicine, Chang Gung University, Taipei, Taiwan, Republic of China; 3 Departments of Physiology and Biophysics, National Defense Medical Center, Taipei, Taiwan, Republic of China; 4 Graduate Institute of Life Sciences, National Defense Medical Center, Taipei, Taiwan, Republic of China; Martin Luther University, Germany

## Abstract

Traumatic brain injury (TBI) triggers a series of neuroinflammatory processes that contribute to evolution of neuronal injury. The present study investigated the neuroprotective effects and anti-inflammatory actions of berberine, an isoquinoline alkaloid, in both *in vitro* and *in vivo* TBI models. Mice subjected to controlled cortical impact injury were injected with berberine (10 mg·kg^−1^) or vehicle 10 min after injury. In addition to behavioral studies and histology analysis, blood-brain barrier (BBB) permeability and brain water content were determined. Expression of PI3K/Akt and Erk signaling and inflammatory mediators were also analyzed. The protective effect of berberine was also investigated in cultured neurons either subjected to stretch injury or exposed to conditioned media with activated microglia. Berberine significantly attenuated functional deficits and brain damage associated with TBI up to day 28 post-injury. Berberine also reduced neuronal death, apoptosis, BBB permeability, and brain edema at day 1 post-injury. These changes coincided with a marked reduction in leukocyte infiltration, microglial activation, matrix metalloproteinase-9 activity, and expression of inflammatory mediators. Berberine had no effect on Akt or Erk 1/2 phosphorylation. In mixed glial cultures, berberine reduced TLR4/MyD88/NF-κB signaling. Berberine also attenuated neuronal death induced by microglial conditioned media; however, it did not directly protect cultured neurons subjected to stretch injury. Moreover, administration of berberine at 3 h post-injury also reduced TBI-induced neuronal damage, apoptosis and inflammation *in vivo*. Berberine reduces TBI-induced brain damage by limiting the production of inflammatory mediators by glial cells, rather than by a direct neuroprotective effect.

## Introduction

Traumatic brain injury (TBI) triggers a series of neuroinflammatory processes that contribute to neuronal injury and failure of functional recovery . Post-traumatic inflammation is mediated by the activation of microglia and astrocytes and infiltration of circulating leucocytes into the affected area. Activated glia produce multiple pro-inflammatory mediators, including cytokines, chemokines, inducible nitric oxide synthase (iNOS) and cyclooxygenase-2 (COX-2). Overproduction of these mediators is toxic to neighboring neurons, which further activates glial cells and injures the remaining neurons through positive feedback [1], [2]. Neurotoxic proinflammatory cytokines can activate receptor-dependent apoptotic pathways via recruitment of adaptor molecules and caspase-8 or -10 activation [3], [4]. Thus, inhibition of glial activation and, therefore, production of inflammatory mediators may be a potential therapeutic strategy for protecting the damaged brain in TBI. Although a number of drugs targeting inflammatory pathways following TBI have been tested in clinical trials, none has conferred a significant benefit [5].

Berberine, an isoquinoline alkaloid isolated from medicinal herbs frequently used in traditional Eastern medicine, has multiple therapeutic effects for metabolic disorders, microbial infection, neoplasms and inflammation [6]. Increasing interest has focused on its anti-inflammatory effects. In microglia, berberine suppresses neuroinflammatory responses [7], [8] and attenuates the production of inflammatory mediators through suppression of toll-like receptor 4 (TLR4)-nuclear factor-κB (NF-κB) signaling in animal models of endotoxemia [9], [10]. Substantial evidence also shows that berberine exerts neuroprotection in cerebral ischemia [11]–[15] and Alzheimer's disease [16]. However, the therapeutic effect of berberine on TBI has yet to be evaluated, and the effective time at which berberine can be administered post-injury has not been investigated in preclinical studies as previous studies have administered it either prior to or shortly after onset [11]–[15].

The aim of the present study was to determine the effects of berberine in a mouse model of TBI. We also examined whether it attenuated TBI-induced glial activation, thereby preventing neuronal damage in both cell and animal models.

## Material and Methods

### Animals

Male C57BL/6J mice (age 8–10 weeks, weight 23–28 g) were obtained from National Laboratory Animal Center (Taipei, Taiwan). All study protocols were approved by the Animal Research Committee at Cheng Hsin General Hospital (Animal permit number CHGH-97-02), and were conducted according to the Guide for the Care and Use of Laboratory Animals published by the US National Institutes of Health (NIH Publication No. 85–23, revised 1996). The results of all studies involving animals are reported in accordance with the ARRIVE guidelines for reporting experiments involving animals [17].

### Experimental protocol

All animals were randomized into one of the three following groups by using computer-generated random numbers: (i) sham injury, (ii) controlled cortical impact (CCI) + vehicle, and (iii) CCI +10 mg·kg^−1^ berberine. All measurements described below were also performed in a blinded manner. Berberine (Sigma, St. Louis, MO, USA) dissolved in 0.9% saline (0.2 mL) or a corresponding volume of vehicle (0.9% saline) was administered intraperitoneally 10 min following injury.

The following measurements were assessed at the indicated time points following injury: 1) behavioral testing at days 1, 4, 7, 14, 21, and 28 (n = 12/group); 2) cresyl violet staining at 2 h, days 1 and 28 (n = 6–8 mice/group); 3) histology, brain water content, Evans blue (EB) dye extravasation, matrix metalloproteinase (MMP)-9 zymography, Western blot analysis, and enzyme-linked immunosorbent assay (ELISA) at 6 h, 12 h, day 1 or day 4 (n = 5–7 mice/group); and 4) real–time quantitative reverse transcription polymerase chain reaction (RT-PCR) at 6 h (n = 7 mice/group). The dose and route of berberine were selected based on previous work in experimental cerebral ischemia [11], [14] and our pilot study in which concentrations of 5, 10, and 15 mg·kg^−1^ were tested; both 10 mg·kg^−1^ and 15 mg·kg^−1^ improved behavioral deficits but there was no significant difference between the two groups (S1 Fig.).

Another set of experiments was performed to investigate the delayed therapeutic potential of berberine for TBI. Berberine or vehicle was delivered intraperitoneally at 3 h post-injury, and the protective effects were assessed using cresyl violet staining, Fluoro-Jade B (FJB) histology, cleaved caspase-3 Western blot (n = 6 mice/group) and ELISA at day 1 (n = 7 mice/group).

### Controlled cortical impact injury

The CCI model was employed to induce TBI as previously described [18]. Briefly, mice were anesthetized with intraperitoneal injection of sodium pentobarbital (65 mg·kg^−1^; Rhone Merieux, Harlow, UK), and a 5-mm craniotomy was performed over the right parietal cortex. CCI was produced using a pneumatic piston with a 2.5 mm-rounded metal tip, velocity of 4 m/sec and depth of 2 mm. The bone flap was immediately replaced and sealed, and the scalp was sutured closed. Body temperature was monitored throughout the surgery by using a rectal probe; temperature was maintained at 37.0±0.5°C using a heated pad. Mice were placed in a heated cage to maintain body temperature while recovering from anesthesia.

Sham-operated animals underwent the same procedure as the injured mice with the exception of CCI.

### Metabolic characteristics assessment

Mice were anesthetized with an overdose of sodium pentobarbital (80 mg·kg^−1^, ip), and right atrial puncture was performed to collect venous blood. The collected blood was centrifuged (3500 g for 5 min), and the serum was stored on ice until analysis. Serum blood urea nitrogen (BUN), creatinine (CRE), and alanine aminotransferase (ALT) were measured by a chemistry autoanalyzer (Synchron Clinical System LX20; Beckman Coulter, Fullerton, CA) to assess renal and liver functions.

### Behavioral testing

Behavioral testing was conducted prior to and at 1, 4, 7, 14, 21, and 28 days after CCI. Animals were pre-trained for 3 days for both the rotarod and beam walking tests.

For the rotarod test, an accelerating rotarod was employed to assess motor function and balance in mice as previously described [19]. The rotarod speed was slowly increased from 6 to 42 rpm within 7 min, and the time during which the animals remained on the rotarod was recorded.

The beam walking test assessed fine motor coordination and function by measuring the ability of the animals to traverse an elevated narrow beam as described previously [19]. The time for the mouse to cross the beam (not to exceed 60 s) was recorded. For the rotarod and beam walking tests, three measurements per trial were recorded 1 h before CCI (baseline) and at each tested time-point after CCI.

The modified neurological severity score (mNSS) is a composite index of motor, sensory, reflex, and balance tests. One point was given for the inability to perform each test or for the absence of a reflex. Neurological function was graded on a scale of 0–18 where a normal score was 0, and a maximal deficit score was 18.

### Histology and immunohistochemistry analyses

Mice were terminally anaesthetized with sodium pentobarbital (80 mg·kg^−1^, ip) and perfused transcardially with saline followed by 4% paraformaldehyde in 0.1 M phosphate buffer. Brain specimens were processed as previously described [19]. Frozen sections (10 µm) were stained with cresyl violet, FJB (Chemicon, Temecula, CA, USA), a marker of degenerating neurons, and terminal deoxynucleotidyl transferase-mediated dUTP-biotin nick end labeling (TUNEL; In situ Cell Death Detection Kit, Roche Molecular Biochemicals, Mannheim, Germany) [20]. For immunostaining, sections were incubated with the respective primary antibody (rabbit polyclonal anti-myeloperoxidase [MPO], a neutrophil marker [Dako, Cambridge, UK], or rabbit anti-ionized calcium binding adaptor molecule 1 [Iba1], a microglia/macrophage marker [Wako Pure Chemical Industries, Osaka, Japan], or CD45, a marker for blood-born monocytes [BD Biosciences Pharmigen, San Jose, CA, USA]). Antibody information is listed in Table 1. The specificity of staining reaction was assessed in several control procedures, including omission of the primary antibody and substitution of the primary antibody with nonimmune rabbit or rat serum.

**Table 1:** Antibodies used in immunofluorescence and western blot.

Primary antibody	Commercial source	Catalog number	Species	Antibody type	Working concentration
Cleaved caspase-3	Cell signaling	9661	Rabbit	Polyclonal	WB 1∶1000
Phospho-Akt Ser473	Cell signaling	9271	Rabbit	Polyclonal	WB 1∶1000
Phospho-Akt Thr308	Cell signaling	4056	Rabbit	Monoclonal	WB 1∶1000
Total Akt	Cell signaling	9272	Rabbit	Polyclonal	WB 1∶2000
Phospho-Bad Ser136	Cell signaling	4366	Rabbit	Monoclonal	WB 1∶1000
Phospho-Erk p44/42	Cell signaling	9101	Rabbit	Polyclonal	WB 1∶1000
Total Erk 44/42	Cell signaling	9102	Rabbit	Polyclonal	WB 1∶2000
TLR4	Santa cruz	Sc-10741	Rabbit	Polyclonal	WB 1∶1000
MyD88	Abcam	2064	Rabbit	Polyclonal	WB 1∶1000
P65	Santa cruz	Sc-372	Rabbit	Polyclonal	WB 1∶1000
iNOS	Cayman	160862	Rabbit	Polyclonal	WB 1∶500
COX-2	Cayman	360106	Mouse	Polyclonal	WB 1∶500
Lamin A/C	Santa cruz	Sc-20681	Rabbit	Polyclonal	WB 1∶1000
Claudin-5	Invitrogen	34-1600	Rabbit	Polyclonal	WB 1∶1000
ZO-1	Invitrogen	61-7300	Rabbit	Polyclonal	WB 1∶200
ICAM-1	Abcam	ab124759	Rabbit	Polyclonal	WB 1∶1000
MPO	Dako	A0398	Rabbit	Polyclonal	IHC 1∶1000
Iba-1	Wako	019-19741	Rabbit	Polyclonal	IHC 1∶1000
CD45	BD	550539	Rat	Monoclonal	IHC 1∶100

### Contusion volume and ventricular enlargement assessment

Contusion volumes and ventricular enlargement ratios were quantified using the cresyl violet-stained sections at 20 rostral-caudal levels that were spaced 200 µm apart as previously described [20]. Sections were analyzed using the ImageJ vision 1.46 software (National Institutes of Health, Bethesda, MD, USA). The volume measurement was computed by summation of the areas multiplied by the interslice distance (200 µm). The ventricular enlargement ratio was expressed as volume ratio (ipsilateral vs. contralateral) [21]. Analysis was performed by two researchers who were blinded to all animal groups. Inter-rater reliability was within 10%.

### Quantification of FJB, TUNEL, MPO, Iba-1, and CD45 staining

FJB, TUNEL, MPO, Iba-1, and CD45 staining was quantified by analyzing three sections per animal at the central lesion level (bregma 0.74 mm). The number of positive cells was counted in an area of 920×860 µm^2^ in 8–10, non-overlapping fields immediately adjacent to the cortical contusion margin using a magnification of ×200. The total number of FJB-, MPO-, Iba1-, and CD45-positive cells was expressed as cells/field. Quantification of TUNEL staining was expressed as (TUNEL-stained nuclei/DAPI-stained nuclei) ×100%. Iba-1-positive resting microglia/macrophages were defined as resting if they contained a relatively small cell body (<7.5 µm in diameter) with long slender processes [22]. Microglia were defined as activated when the cell body was increased in size compared to resting microglia with short, thick processes and intense immunointensity. Activated microglia were defined based on a combination of morphological criteria and a cell body diameter cutoff of 7.5 µm.

Analysis was conducted by two independent researchers who were blinded to all animal groups. Inter-rater reliability was within 10%.

### Brain water content

Brain water content represents brain edema, which forms as a consequence of Blood-brain barrier (BBB) breakdown and post-injury inflammation. Mice were terminally anaesthetized with sodium pentobarbital (80 mg·kg^−1^, ip) and sacrificed by decapitation at day 1 post-injury. The cerebellum (internal control) and cortex of each hemisphere were weighed (wet weight), dried at 100°C for 24 h, and reweighed (dry weight). Water content was determined as [(wet weight-dry weight)/wet weight] ×100% (Chang *et al.*, 2011).

### Evaluation of blood-brain barrier permeability

BBB permeability was evaluated by measuring EB extravasation as previously described [19]. Briefly, EB dye (4 mL·kg^−1^ in 2% saline) was administered via the tail vein and allowed to circulate for 60 min. Brains were removed, and ipsilateral hemispheres were cut into 4-mm-thick sections (2 mm from the frontal pole) and weighed. For the extraction of EB from brain tissues, hemispheres were placed in 1 mL of 60% trichloroacetic acid and homogenized by sonication. The absorbance of each supernatant for the EB dye was measured at 620 nm using a spectrophotometer. EB concentrations were calculated and expressed as µg·g^−1^ brain tissue against a standard curve.

### Western blot analysis

Mice were terminally anaesthetized with sodium pentobarbital (80 mg·kg^−1^, ip ) and sacrificed by decapitation at 6 h, 12 h, or 1 day following CCI or sham surgery. A 4-mm coronal section from the injured area over the right parietal cortex was collected. Mixed glial cultures were collected at 24 h post-stretch injury. Western blot analysis was performed as previously described [23] using the antibodies listed in Table 1. Briefly, equal amounts of protein (35 to 50 µg protein in 20 µl for tissue samples and 20 µg protein in 20 µl for cell lysates) were separated by sodium dodecyl sulfate-polyacrylamide gel, transferred to Immobilon-P membranes (Millipore, Billerica, MA, USA), blocked using 5% milk in PBS containing 0.1% Tween-20, and probed with primary antibodies at 4°C overnight. Afterwards, the membranes were washed and incubated with horseradish peroxidase-linked anti-rabbit or anti-mouse secondary antibodies (1∶1000 dilution; Santa Cruz Biotechnology) for 2 h. The relative intensity of each protein signal was normalized to the corresponding β-actin intensity and quantified by densitometry analysis using Image J software.

### Real-time quantitative RT-PCR

Total RNA was extracted for reverse transcription, and real-time quantitative RT-PCR analysis was performed on an ABI PRISM 7900 sequence detector (Applied Biosystems, Foster City, CA, USA) as previously described [23]. Primers and probes for interleukin (IL)-1β (TaqMan Gene Expression Assay ID Mm00434228_ml), IL-6 (Mm00446190_ml), monocyte chemoattractant protein-1(MCP-1, Mm00441242_m1), and macrophage inflammatory protein-2 (MIP-2, Mm00436450_m1) were purchased from Applied Biosystems. β-Actin (Rn00607939_s1) was used as endogenous control. Thermal cycling was initiated with a 2-min incubation at 50°C, followed by a 10-min denaturation step at 95°C and 40 cycles at 95°C for 15 s and 60°C for 1 min. Relative quantities of the candidate genes and β-actin mRNA were calculated using the comparative threshold cycle (Ct) method.

### ELISA and nitrite assay

ELISA for interleukin IL-1β, IL-6, MCP-1, and MIP-2 levels in brain homogenates or cell lysates was performed using commercially available kits (R&D Systems, Minneapolis, MN, USA). All samples and standards were assayed in duplicate according to the manufacturer's instructions. Nitric oxide (NO) production was assessed by measuring the nitrite levels in the culture supernatants with the Griess reagent (Sigma-Aldrich; St. Louis, MO, USA).

### Gelatin zymography

Zymography was performed as previously described [23]. Briefly, equal amounts of protein were separated on a 10% Tris-glycine gel copolymerized with 0.1% gelatin as substrate. After separation, the gel was washed twice in distilled water (30 min each wash) and then, proteins within the gel were renatured by incubation with 2.5% Triton-X-100 buffer for 1 h at room temperature. After incubating with developing buffer (0.05 M Tris-HCl pH 7.5, 0.2 M NaCl, 5 mM CaCl_2_, 0.05% Brij-35, and 0.2 mM NaN_3_) at 37°C for 24 h, the gel was stained with 0.05% Coomassie R-250 dye (Sigma) for 30 min followed by destaining. The recombinant mouse MMP -9 (Abcam plc, Cambridge, UK) was used as a positive control to identify the active form of MMP-9. Gelatinolytic activity (MMP-9: ∼89 kDa) was determined as clear bands at the appropriate molecular weight.

### Preparation of primary cortical neuron, mixed glial and microglial cultures and induction of neuron stretch injury

Primary cortical neuron cultures were prepared from embryonic C57BL/6 mice at 15.5 days and used at day 10 *in vitro* as previously described [23]. Cortical neurons were stretched by rapid deformation of the silastic culture plates using the Cell Injury Controller II (Custom Design and Fabrication; Virginia Commonwealth University, Richmond, VA, USA) as previously described [20]. The injury controller delivered one 50-ms pulse (28 psi) of compressed nitrogen, which resulted in a 10.2 psi peak pressure to the well and deformed the membrane 6.5 mm. The primary cultured neurons were rapidly stretched 135% of their original length and were treated with various concentration of berberine immediately post-injury. Cell viability was assessed 24 h after stretch injury using the 3-[4,5-dimethyl-2-thiazolyl]-2,5-diphenyl-2-tetrazolium bromide (MTT) reduction assay. Cells were incubated at 37°C for 2 h with MTT (0.5 mg/ml; Sigma-Aldrich), and then a solution of anhydrous isopropanol, HCl (0.1 N), and 0.1% Triton X-100 was added to dissolve the water-insoluble formazan. The optical density was determined at 570 nm. Cell viability was expressed as a percentage of the control culture.

Primary mixed glial cultures were prepared from l-day-old neonatal C57BL/6 mice as described previously with some modifications [24]. In brief, brain cortical tissues were dissociated in Dulbecco's modified Eagle medium (DMEM; Gibco/BRL, Bethesda, MD, USA) supplemented with 10% heat-inactivated fetal bovine serum (FBS; Gibco/BRL), 100 U·mL^−1^ penicillin, and 100 µg mL^−1^ streptomycin and were seeded in 6- or 24-well culture plates. The medium was changed after 5 days and every 3 days thereafter. The cell cultures were used 14 days after plating. The mixed glia cultures contained ∼75–85% astrocytes and ∼15–25% microglia by immunocytochemical staining. Mixed glial cells were stimulated with 10 ng·mL^−1^ IL-1β (R&D Systems) for 24 h in the presence or absence of varying concentrations of berberine.

For pure microglial culture, microglial cells were isolated from culture flasks of confluent glial cultures by shaking at 75 r.p.m. for 4–6 h [25]. Microglial cells in the supernatant were collected by centrifugation at 1200 r.p.m. for 10 min. Purified microglia were seeded into 24-well plates at 1×10^5^ cells mL^−1^. The purity of microglial cultures was greater than 95% as determined by immunohistochemical staining using the microglia-specific marker Iba1 and the astrocyte marker GFAP. Microglial cells were stimulated with 1 µg·mL^−1^ IL-1β for 48 h in the presence or absence of 50 µM berberine. All *in vitro* experiments were repeated four times with different batches of cultures.

### Neuron survival after addition of microglial conditioned medium

The mouse microglial BV2 and neuroblastoma neuro-2A (N2A) cell lines were cultured in DMEM supplemented with 10% heated FBS, 100 U·mL^−1^ penicillin and 100 µg·mL^−1^ streptomycin in a humidified atmosphere of 5% CO_2_ at 37°C. For collection of conditioned media, BV2 microglia were plated and incubated with 1 µg·mL^−1^ IL-1β in the absence (IL-1β–CM) or presence of 50 µM berberine (IL-1β/BBR–CM) for 48 h. Cell-free supernatant fractions were applied to N2A cells for 24 h to evaluate the changes in cell viability and related parameters. Neuronal cell death was assessed by the MTT assay. The experiments were repeated four times with different batches of cultures.

### Statistical analyses

Data are presented as the mean and standard error of the mean (mean ± SEM). One-way or two-way analysis of variance (ANOVA) followed by post-hoc Bonferroni evaluation was used for multiple groups to determine significant differences. Student's *t*-test was used to evaluate differences between two groups. *P* values <0.05 were considered statistically significant.

## Results

### Berberine improved long-term neurobehavioral function after TBI

**Figure 1:**
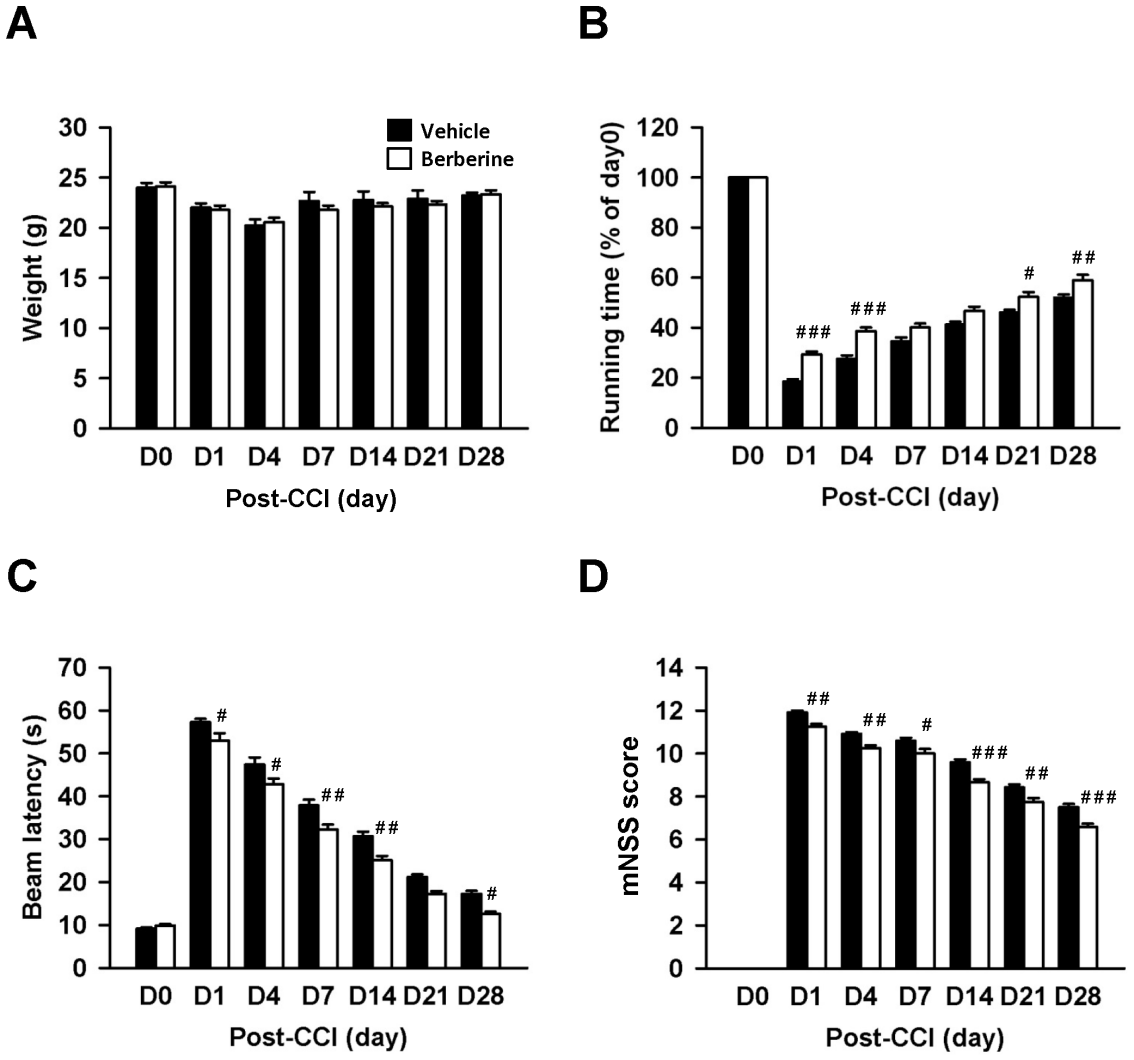
Post-injury berberine treatment improved long-term neurobehavioral functions without affecting body weight. (**A**) There were no significant differences in body weight between the vehicle control and 10 mg·kg^−1^ berberine treatment groups during the 28-day observation period post-TBI. (**B**) Berberine significantly increased the rotarod running time compared with vehicle-treated mice on days 1, 7, 21, and 28 post-CCI. (**C**) Beam walk latencies were significantly shorter for the 10 mg·kg^−1^ berberine group than the vehicle control group at 1, 4, 7, 14, and 28 days post-CCI. (**D**) The mNSSs were significantly lower in the 10 mg·kg^−1^ berberine group than the vehicle group at all time points analyzed after injury. Values are presented as mean ± SEM; ^#^ *P*<0.05, ^##^ *P*<0.01, and ^###^ *P*<0.001 vs. the vehicle control group as determined by two-way ANOVA. (n = 12 mice/group).

To determine the safety of berberine, body weight and mortality were assessed during the 28-day observation period. There was no difference in body weight (*P*>0.05; Fig. 1A) or 28-day mortality, which was lower than 10% in both groups (*P*>0.05, data not shown), between vehicle and berberine-treated groups. Also, serum levels of BUN, CRE, and ALT did not differ between the groups at 1 day post-injury, suggesting no differences in renal or hepatic function between the treatment groups (Table 2).

**Table 2:** Metabolic characteristics of the sham control, mice treated with vehicle and berberine.

	CCI Day 1			
	Sham	Vehicle	Berberine	Reference range
**BUN (mg/dL)**	23.30±0.82	22.55±0.87	22.82±0.71	8–33
**CRE (mg/dL)**	0.17±0.01	0.17±0.02	0.18±0.02	0.2–0.9
**ALT (mg/dL)**	23.5±1.65	25.33±1.15	22.83±0.87	17–77

Values are expressed as means ± SEM. n = 6 mice/group. CCI: cortical impact injury; BUN: blood urea nitrogen; CRE: creatinine; ALT: alanine aminotransferase;

To explore the neuroprotective potential of berberine for TBI, we first evaluated motor and neurological function, which are affected in many TBI patients who fail to ambulate or perform self-care independently. TBI induced a significant impairment in both rotarod and beam walk performance at all tested time points in the vehicle-treated mice (Fig. 1B and C). Treatment with berberine significantly increased the rotarod running time at 1, 4, 21 and 28 days post-injury as compared to vehicle-treated mice (all *P*<0.05; Fig. 1B). Similarly, berberine-treated mice exhibited better beam walk performances with significantly reduced latency to cross the beam from 1 to 28 days after CCI (*P*<0.05 on days 1, 4, and 28; *P*<0.01 on days 7 and 14; Fig. 1C). Moreover, treatment with berberine significantly decreased mNSS compared with the vehicle group from days 1 to 28 post-injury (all *P*<0.05; Fig. 1D). These data suggest that a single injection of berberine is sufficient to improve long-term functional outcomes after TBI.

### Berberine attenuated contusion volumes, neuronal death and apoptosis after TBI

**Figure 2:**
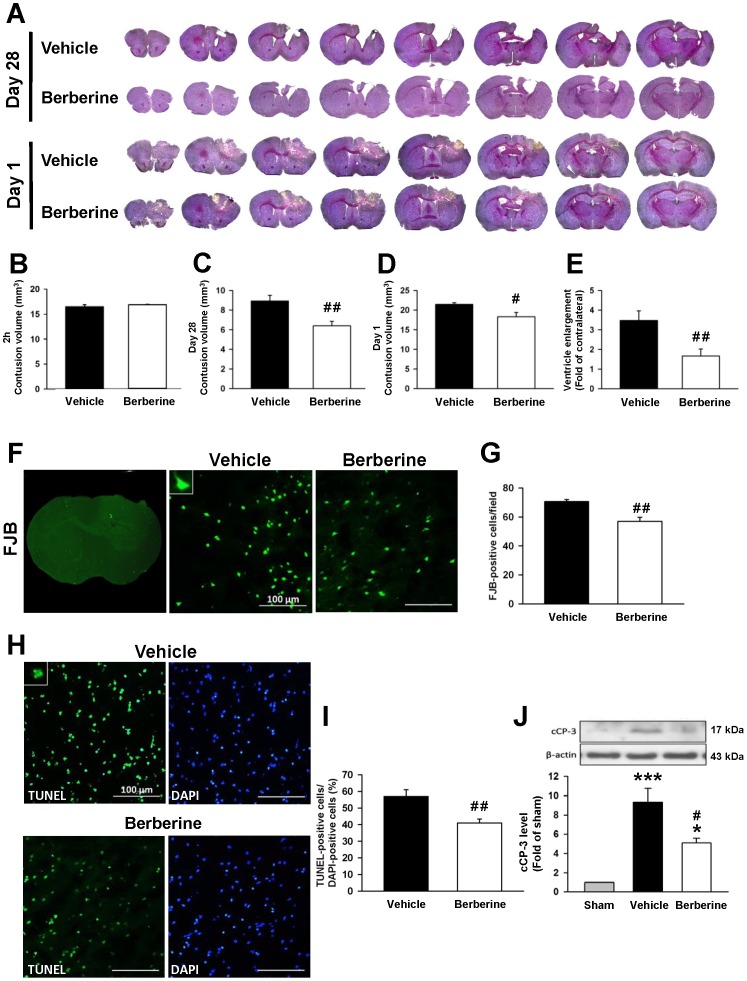
Post-injury berberine treatment attenuated contusion volume, neuronal death and apoptosis after TBI. (**A**) Representative cresyl violet-stained brain sections from vehicle- and 10 mg·kg^−1^ berberine-treated mice at 1 and 28 days post-TBI illustrating hypointense regions immediately below the impact site in the cortex. Quantitative analysis showed that berberine did not affect contusion volumes at (**B**) 2 h but significantly reduced contusion volumes compared with vehicle-treated mice at both (**C**) 28 and (**D**) 1 days post-TBI. (**E**) Lateral ventricular enlargement was significantly smaller in 10 mg·kg^−1^ berberine-treated mice than in vehicle-treated mice on day 1. (**F**) Representative Fluoro-Jade B (FJB)-stained coronal section of a core contusion region 0.74 mm from the bregma. The inset is a representative FJB-positive cell at higher magnification. (**G**) Quantitative analysis indicates that berberine-treated mice had significantly fewer degenerating neurons than vehicle-treated mice in the cortical contusion margin at 1 day post-TBI. The total number of FJB-positive cells is expressed as the mean number per field of view (0.8 mm^2^). Scale bar, 100 µm. (**H**) Representative terminal deoxynucleotidyl transferase-mediated dUTP-biotin nick end labeling (TUNEL, green)- and DAPI (blue)-stained brain sections of a 10 mg·kg^−1^ berberine- and vehicle-treated mouse at 1 day post-TBI. The inset is a representative TUNEL-positive cell at higher magnification. (**I**) Quantitative analysis shows that berberine-treated mice had significantly fewer TUNEL-positive cells than the vehicle-treated mice in the cortical contusion margin at 1 day post-TBI. The percentage of TUNEL-positive cells is expressed as the number of TUNEL-stained nuclei/the total number of DAPI-stained nuclei. Sections were stained with DAPI (blue) to show all nuclei. Scale bar, 100 µm. Values are mean ± SEM: ^#^ *P*<0.05 and ^##^ *P*<0.01 vs. the vehicle group as determined by the Student's *t*-test (n = 6–8 mice/group for cresyl violet and n = 6 mice/group for FJB and TUNEL histochemistry). (**J**) Western blot analysis of cleaved caspase-3 (cCP-3) in the ipsilateral hemisphere of sham-injured, vehicle-treated, and 10 mg·kg^−1^ berberine-treated mice at 1 day post-TBI. Berberine significantly decreased the cCP-3 level. Values are mean ± SEM; ^*^ *P*<0.05, ^***^ *P*<0.001 vs. the sham group; ^#^ *P*<0.05 vs. the vehicle group as determined by one-way ANOVA (n = 6–8 mice/group for cCP-3 Western blot).

To determine whether the improvement in neurobehavioral recovery with berberine reflects a reduction in tissue damage and neuronal death, we next measured the extent of brain tissue damage and neuronal injury. At 2 h after injury, there was no difference in contusion volume between vehicle-treated and berberine-treated mice, indicating that brain damage was the same initially regardless of treatment (Fig. 2A and B). However, as shown in Fig. 2C–E, berberine significantly reduced contusion volume by approximately 28% compared to vehicle (6.4±0.5 vs. 8.9±0.6 mm^3^; *P* = 0.0034) at 28 days post-injury. CCI increased ipsilateral lateral ventricle size by 3.5±0.5-fold relative to their contralateral hemisphere in the vehicle group, indicating tissue loss in the ipsilateral hemisphere. Berberine attenuated ventricle size by 1.7±0.4-fold (*P* = 0.0065).

Chronic tissue loss following TBI was associated with initial neuronal death in our previous study [20]. To evaluate whether berberine attenuates neurodegeneration at the acute stage, we analyzed brain tissue damage, neuronal damage and apoptosis at 1 day post-injury. Consistent with the neuroprotective effect at 28 days, berberine significantly reduced contusion volume compared to vehicle (18.3±1.1 vs. 21.4±0.4 mm^3^; *P* = 0.0106; Fig. 2D) at 1 day. Similarly, the number of FJB-positive degenerative neurons around the injured cortical area was significantly decreased in berberine-treated animals compared to vehicle-treated animals (56.9±2.8 vs. 70.6±1.4 cells/field; *P* = 0.008; Fig. 2F and G). Moreover, TUNEL-positive nuclei were observed in the ipsilateral injured cortex but not in the contralateral side at 1 day post-injury (Fig. 2H); however, the berberine-treated mice had significantly fewer TUNEL-positive cells around the injured cortical areas than the vehicle group (40.9±2.4% vs. 56.8±4.0%; *P* = 0.0031; Fig. 2H and I). Cleaved caspase-3, a critical effector caspase in apoptosis, was also reduced by 45.7% following berberine treatment compared to the vehicle group at 1 day post-injury (*P*<0.05; Fig. 2J).

### Berberine did not alter PI3K/Akt or Erk signaling

**Figure 3:**
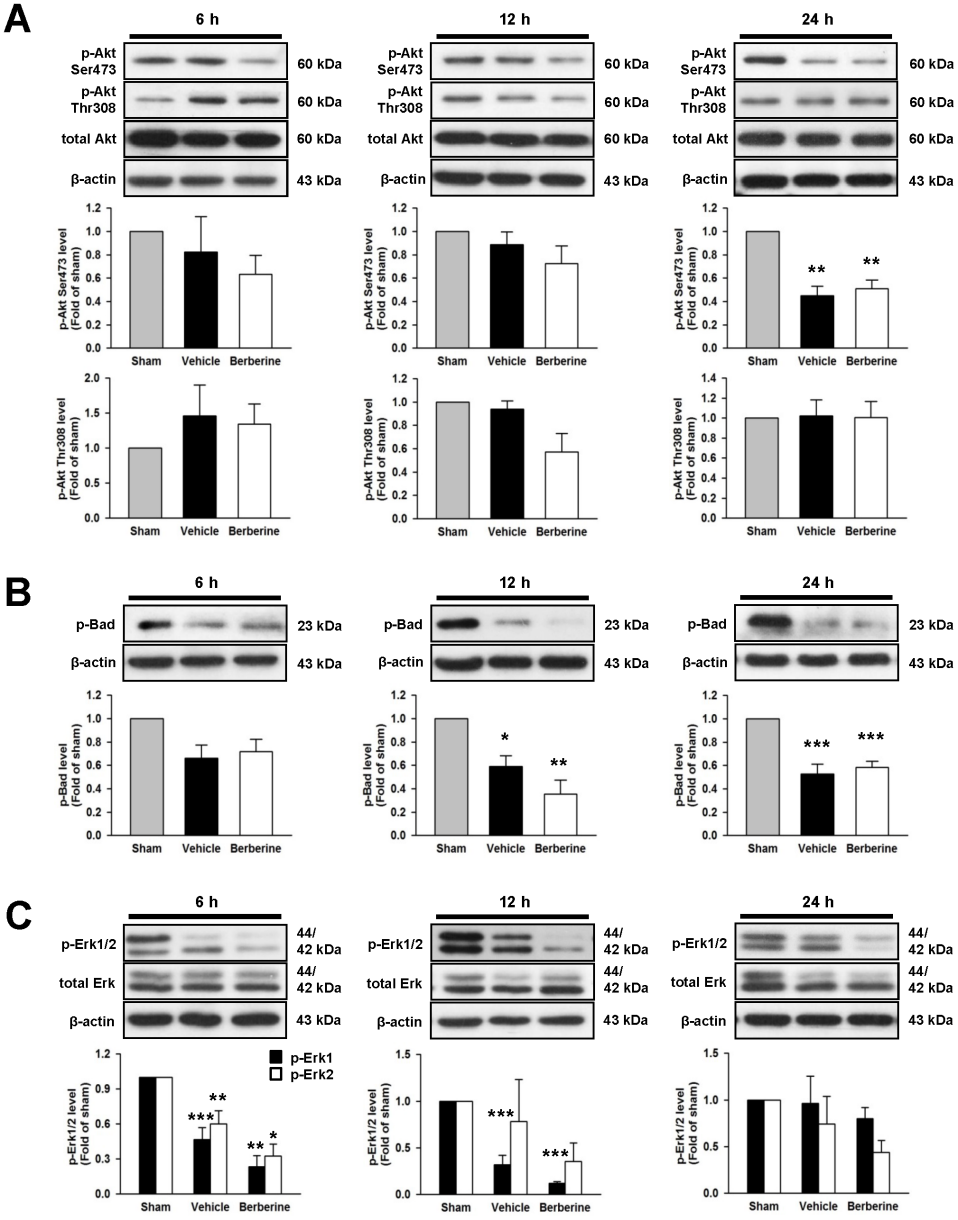
Post-injury berberine treatment did not affect PI3K/Akt or Erk signaling after TBI. Western blot analysis of (**A**) phospho-Akt, (**B**) phospho-Bad, and (**C**) phospho-Erk levels in the ipsilateral hemisphere of sham-injured, vehicle-treated, and 10 mg·kg^−1^ berberine-treated mice at 6 h, 12 h, and 1 day post-TBI. Berberine did not affect the phosphorylation of Akt at Ser473 or Thr308, Bad (the downstream factor of Akt) or Erk 1/2 at any tested time-points. Values are mean ± SEM; ^*^ *P*<0.05, ^**^ *P*<0.01, ^***^ *P*<0.001 vs. The sham group as determined by one-way ANOVA (n = 5–7 mice/group for all Western blot analysis).

To examine whether the decrease in FJB-positive and TUNEL-positive cells observed in berberine-treated mice was due to activation of survival signaling pathways, we examined the PI3K/Akt and Erk pathways, which regulate neuronal survival [26]. CCI induced a significant decrease in Akt Ser473 phosphorylation levels at 1 day post-injury (*P*<0.001); however, Akt Thr308 phosphorylation levels remained unchanged at all time points analyzed (all *P*>0.05; Fig. 3A). Neither Akt Ser473 nor Thr308 phosphorylation was altered with berberine treatment (all *P*>0.05; Fig. 3A). In addition, berberine treatment did not alter Bad phosphorylation levels, a downstream mediator of Akt signaling (Fig. 3B). Likewise, the levels of Erk1/2 proteins following CCI were similar between the vehicle and berberine treatments groups (all *P*>0.05; Fig. 3C). These findings demonstrate that berberine did not alter PI3K/Akt or Erk signaling.

### Berberine attenuated brain edema, BBB permeability, MMP-9 enzymatic activity and neutrophil infiltration after TBI

**Figure 4:**
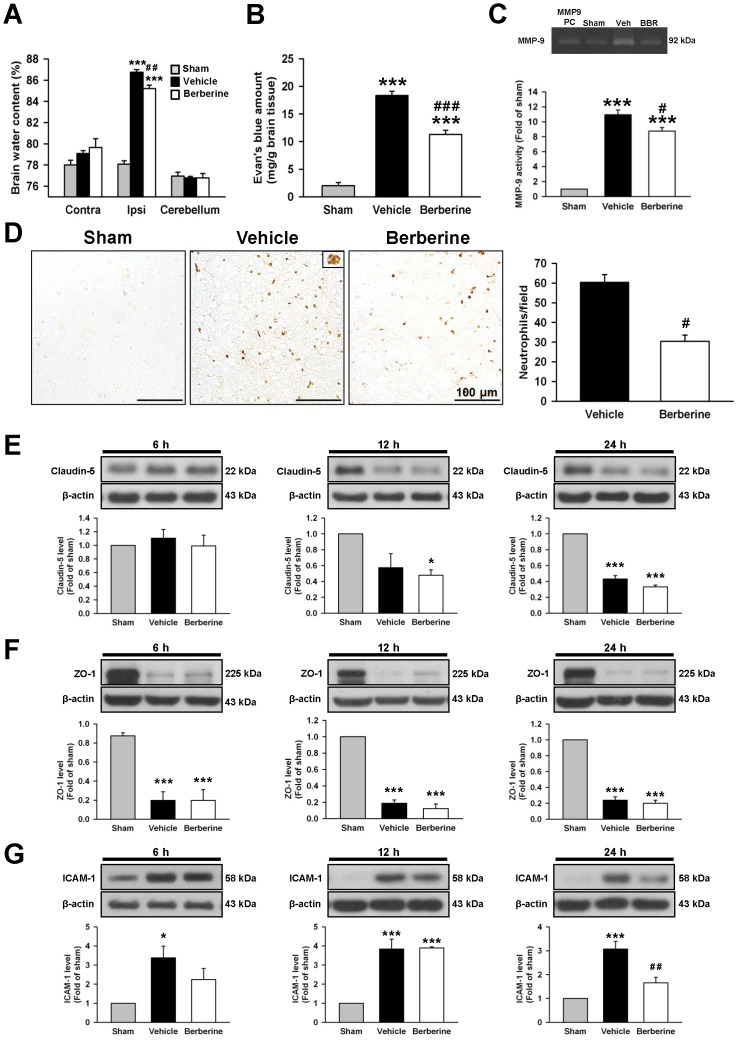
Post-injury berberine treatment attenuated brain edema, blood-brain barrier (BBB) permeability, matrix metalloproteinase (MMP)-9 enzymatic activity, neutrophil infiltration and ICAM expression after TBI. Berberine (10 mg·kg^−1^) significantly decreased (**A**) brain water content and (**B**) leakage of Evans Blue into the brain and in the ipsilateral hemisphere compared with the vehicle-treated mice. (**C**) Representative zymography of MMP-9 activity from a sham-injured control, a 10 mg·kg^−1^ berberine-treated, and a vehicle-treated mouse at 1 day post-TBI including the MMP-9 standard as a positive controls (PC). The gelatinase activity of MMP-9 was significantly decreased in berberine-treated mice compared with vehicle-treated mice. (**D**) Representative myeloperoxidase (MPO)-stained brain sections from a sham-injured control, a 10 mg·kg^−1^ berberine-treated, and a vehicle-treated mouse at 1 day post-TBI. The inset is a representative MPO-positive cell at higher magnification. Cell count analysis shows that berberine-treated mice had significantly fewer infiltrating neutrophils than vehicle-treated mice in the cortical contusion margin at 1 day post-TBI. The number of MPO-positive cells is expressed as the mean number per field of view (0.8 mm^2^). Scale bar, 100 µm. Western blot analysis of (**E**) claudin-5, (**F**) zonula occludens 1 (ZO-1), and (**G**) intercellular adhesion molecule (ICAM)-1 levels in the ipsilateral hemisphere of sham-injured, vehicle-treated, and 10 mg·kg^−1^ berberine-treated mice at 6 h, 12 h, and 1 day post-TBI. Treatment with 10 mg·kg^−1^ berberine did not affect TBI-mediated reduced expression of claudin-5 or ZO-1 at any tested time-points but attenuated ICAM-1 expression at 1 day. Values are mean ± SEM; ^*^ *P*<0.05, ^***^ *P*<0.001 vs. the sham group; ^#^ *P*<0.05, ^##^ *P*<0.01 vs. the vehicle group as determined by one-way ANOVA (n = 5–7 mice/group for brain water content, Evans Blue amount, MMP-9 activity and n = 4–6 mice/group for all Western blot analysis). Values are presented as means ± SEM; ^#^ *P*<0.05 vs. the vehicle group as determined by the Student's *t*-test (n = 5–6 mice/group for MPO staining).

We next explored whether berberine directly influenced post-injury inflammatory events by first measuring brain edema and BBB breakdown at 1 day post-CCI as both are a consequence of post-injury inflammation [27], and brain edema is reported to peak at 1 day post-CCI [28]. As shown in Fig. 4A, brain water content was significantly increased in the ipsilateral hemisphere in the vehicle group compared to the sham-operated group (86.7±0.3% vs. 78.1±0.3%; *P*<0.001), which was significantly decreased with berberine treatment (85.2±0.3% vs. 86.7±0.3%; *P*<0.01). Analysis of BBB permeability, as determined by leakage of albumin-bound EB into the brain, revealed that CCI significantly increased EB leakage in the ipsilateral hemisphere compared with the sham-control at day 1 (18.4±.8 vs. 2.0±0.6 µg/g, *P*<0.001), which was attenuated with berberine (11.3±0.8 vs. 18.4±0.8 µg/g, *P*<0.001; Fig. 4B).

We next investigated whether administration of berberine following TBI reduced neutrophil infiltration and macrophage activation, both of which contribute to BBB breakdown and brain edema [27]. Analysis of MMP-9, which can mediate BBB breakdown by degrading the basal lamina [27] and contribute to the pathophysiology of TBI [29] revealed that its activity was significantly increased 1 day post-injury and was significantly attenuated with berberine (*P*<0.05; Fig. 4C). CCI induced a robust infiltration of neutrophils in the injured cortex while berberine significantly reduced neutrophil accumulation in the pericontusion area compared with vehicle-treated mice (30.5±3.1 vs. 60.3±4.0 cells/field, *P*<0.05; Fig. 4D).

We next sought to determine the effects of berberine on two major proteins involved in tight junctions of the BBB, claudin-5 and zonula occludens (ZO)-1, and an endothelial adhesion molecule, intercellular adhesion molecule (ICAM)-1. CCI caused a significant decrease in claudin-5 expression at 1 day post-injury (*P*<0.001; Fig. 4E) and a significant decrease in ZO-1 expression from 6 h to day 1 after injury (all *P*<0.001; Fig. 4F). Neither claudin-5 nor ZO-1 expression was altered with berberine treatment (all *P*>0.05; Fig. 4E & F). Also, CCI caused an upregulation of ICAM-1 in the injured cortices of vehicle-treated mice at all tested time-points (all *P*<0.001; Fig. 4G). However, this increase of ICAM-1 expression was reversed after berberine treatment at day 1. Protein expression of ICAM in the injured cortex of berberine-treated mice was decreased to 54% (*P*<0.01) of the vehicle group.

### Berberine reduced microglial activation, macrophage infiltration and expression of inflammatory cytokines and chemokines after TBI

**Figure 5:**
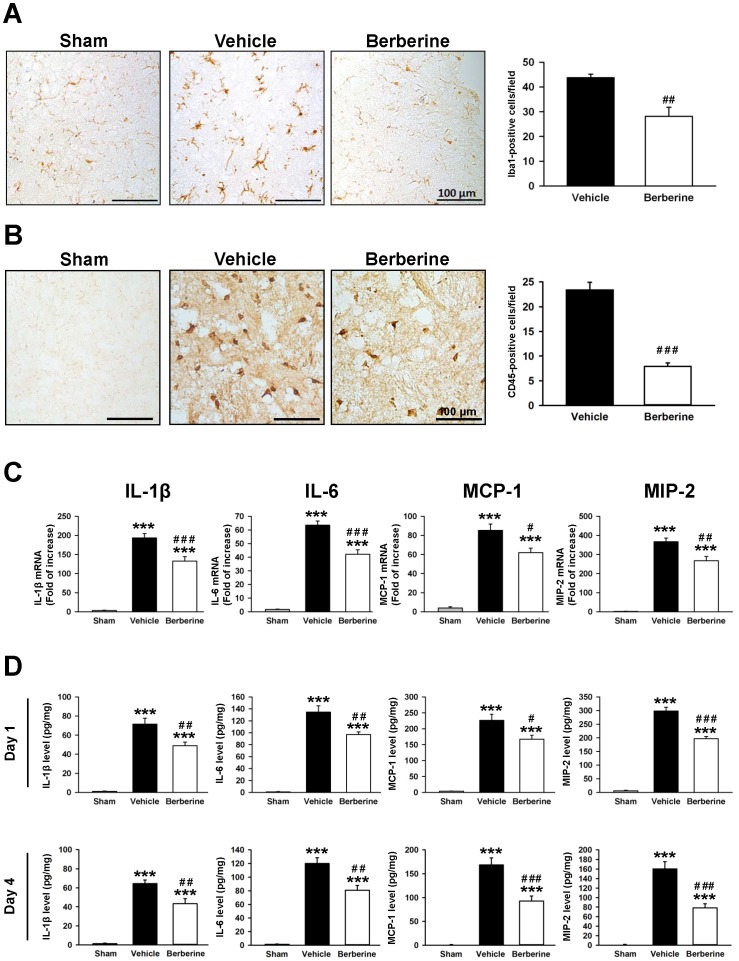
Post-injury berberine treatment reduced microglial activation, macrophage infiltration and expression of inflammatory cytokines and chemokines. Representative (**A**) anti-ionized calcium binding adaptor molecule 1 (Iba1)-stained and (**B**) CD45-stained brain sections from a sham-injured control, a vehicle-treated, and a 10 mg·kg^−1^ berberine-treated mouse at 1 day post-TBI. Cell count analysis shows that berberine-treated mice had significantly fewer Iba1-positive and CD45- positive cells than vehicle-treated mice in the cortical contusion margin at 1 day post-TBI. The number of Iba1-positive and CD45-cells is expressed as the mean number per field of view (0.8 mm^2^). Scale bar, 100 µm. Values are presented as means ± SEM; ^##^ *P*<0.01 vs. the vehicle group as determined by the Student's *t*-test (n = 6 mice/group for Iba1 and CD45 staining). (**C**) Bar graphs demonstrating interleukin (IL)-1β, IL-6, monocyte chemoattractant protein (MCP)-1, and macrophage inflammatory protein (MIP-2) mRNA expression as assessed by Taqman reverse transcription-polymerase chain reaction in the ipsilateral cortices of sham control, vehicle-treated, and 10 mg·kg^−1^ berberine-treated mice at 6 h post-injury. Berberine significantly attenuated IL-1β, IL-6, MCP-1 and MIP-2 mRNA expression compared with vehicle-treated mice. (**D**) Bar graphs of IL-1β, IL-6, MCP-1, and MIP-2 protein concentrations, as assessed by enzyme-linked immunosorbent assays in the ipsilateral cortices of sham control, vehicle-treated, and 10 mg·kg^−1^ berberine-treated mice at 1 and 4 days post-injury. Berberine-treated mice exhibited significantly reduced IL-1β, IL-6, MCP-1, and MIP-2 protein levels compared with vehicle-treated mice at both 1 day 4 days. Values are presented as means ± SEM; ***P<0.001 vs. the sham control; ^#^ *P*<0.05, ^##^ *P*<0.01, ^###^ *P*<0.001 vs. the vehicle group as determined by one-way ANOVA (n = 5–7 mice/group for ELISA and n = 7 mice/group for RT-PCR).

To determine whether berberine affected microglial activation and the expression of inflammatory mediators, we assessed the mRNA and protein expression levels of various inflammatory mediators at 6 h, 1 and 4 days post-injury given that most cytokines and chemokines peaked at 6 and 24 h after TBI, respectively but declined after 4 days [30]. As shown in Fig. 5A, Iba1-positive activated microglia/macrophages were observed within the injured cortex at 1 day, and the number of active microglia/macrophages was significantly reduced with berberine compared with the control group (47.2±2.2 vs. 33.1±2.9 cells/field, *P*<0.01). We further used CD45 staining to determine whether berberine affected macrophage infiltration. The expression of CD45 is low in resting microglia and increases during microglial activation, whereas CD45 expression is high in blood-born monocytes [31], [32]. Similarly, berberine treatment significantly reduced the number of CD45-positive cells in the injured cortex at 1 day compared with the control group (23.4±1.6 vs. 7.9±0.7 cells/field, *P*<0.001; Fig. 5B). In addition, IL-1β, IL-6, MCP-1 and MIP-2 mRNA levels in the injured cortex were significantly increased in the injured cortices of vehicle-treated mice at 6 h post-injury compared with sham controls (all *P*<0.001), and were significantly attenuated with berberine (Fig. 5C). IL-1β, IL-6, MCP-1, and MIP-2 mRNA levels in the berberine-treated injured cortex were 56.0% (*P*<0.001), 73.5% (*P*<0.001), 54.2% (*P*<0.05), and 47.7% (*P*<0.01) of that observed for the vehicle group, respectively. Berberine also significantly reduced IL-1β, IL-6, MCP-1, and MIP-2 protein levels after CCI as compared with the vehicle group at both 1 day (IL-1β: 48.9±3.8 versus 71.6±6.2 pg·mg^−1^ protein, *P*<0.01; IL-6: 96.9±4.5 versus 134.6±10.6 pg·mg^−1^ protein, *P*<0.01; MCP-1: 167.4±12.4 versus 226.2±19.2 pg·mg^−1^ protein, *P*<0.05; MIP-2: 197.1±8.0 versus 298.8±13.2 pg·mg^−1^ protein, *P*<0.001, Fig. 5D) and 4 days post-injury (IL-1β: 43.2±5.5 versus 64.4±3.8 pg·mg^−1^ protein, *P*<0.01; IL-6: 80.9±7.1 versus 120.0±8.5 pg·mg^−1^ protein, *P*<0.01; MCP-1: 92.7±10.9 versus 168.4±14.9 pg·mg^−1^ protein, *P*<001; MIP-2: 78.5±8.5 versus 160.3±15.1 pg·mg^−1^ protein, *P*<0.001, Fig. 5D).

### Berberine attenuated mixed glia and microglia activation by IL-1β and protected neurons against microglia-mediated neurotoxicity

**Figure 6:**
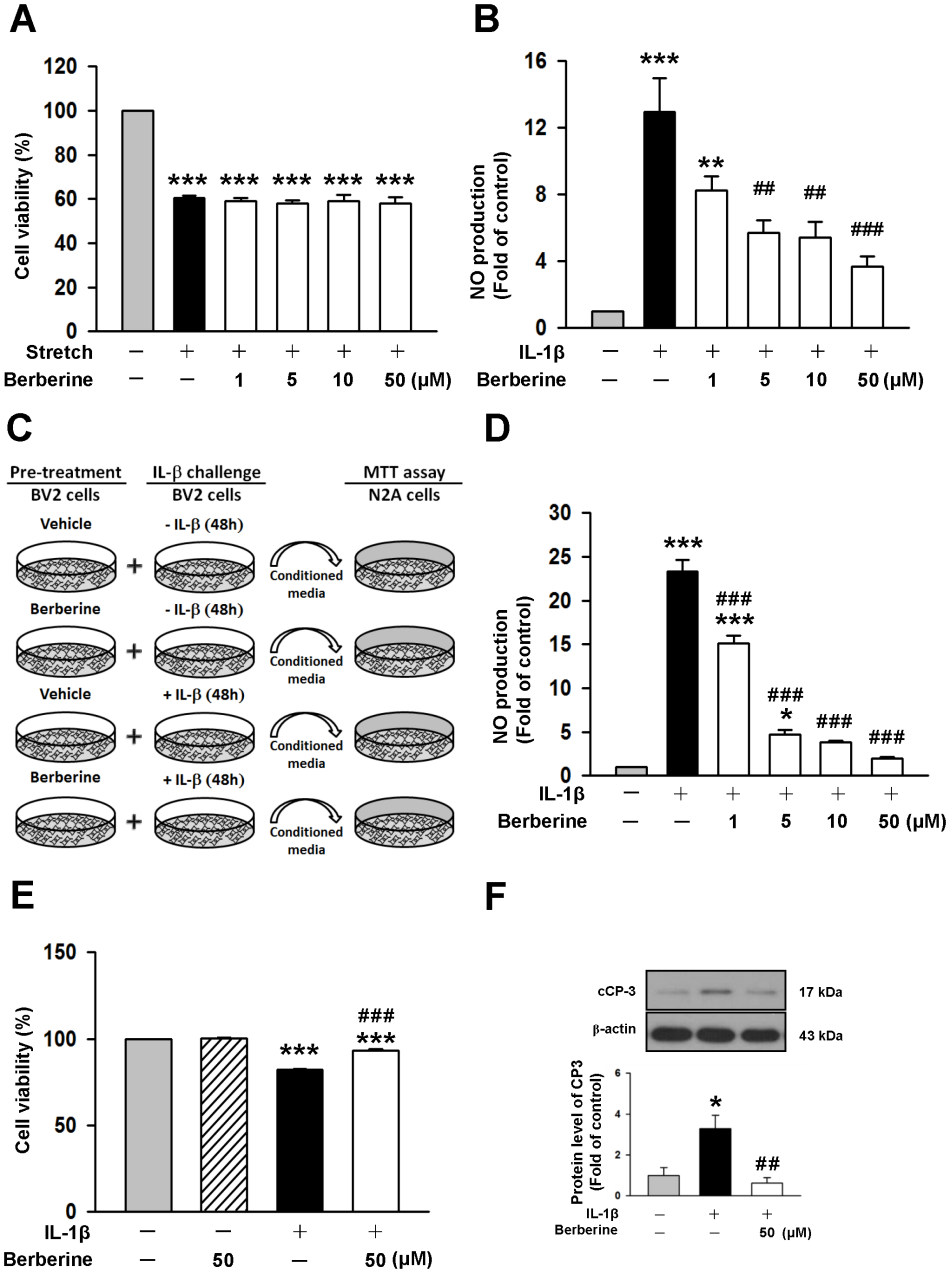
Berberine reduced interleukin-1**β** (IL-1β)-induced activation of mixed glia and microglia and protected neurons against microglia-mediated neurotoxicity *in vitro*. (**A**) Treatment with 1, 5, 10 or 50 µM berberine immediately after stretch injury for 1 day did not affect cell viability by 3-[4,5-dimethyl-2-thiazolyl]- 2,5-diphenyl-2-tetrazolium bromide (MTT) assay. (**B**) In primary mixed glial cultures, co-treatment of 5, 10, or 50 µM berberine with IL-1β for 1 day significantly reduced IL-1β-induced nitric oxide (NO) production. (**C**) Experimental scheme of neuronal survival in neuroblastoma neuro-2A (N2A) cells in response to IL-1β-treated BV2-conditioned media with or without berberine pretreatment. BV2 microglia were incubated with 1 µg·mL^−1^ IL-1β in the absence (IL-1β–CM) or presence of 50 µM berberine (IL-1β/BBR–CM) for 48 h. Cell-free supernatant fractions were applied to N2A cells for 24 h to evaluate the changes in cell viability and cleaved caspase-3 (cCP-3)level. (**D**) In BV2 microglia, co-treatment of 1, 5, 10, or 50 µM berberine with IL-1β for 2 days significantly reduced IL-1β-induced NO production. Values are presented as mean ± SEM of four independent experiments. ^*^ *P*<0.05, ^**^ *P*<0.01, and ^***^ *P*<0.001 vs. the normal control; ^##^ *P*<0.01 and ^###^ *P*<0.001 vs. stretch injury or IL-1β stimulation alone as determined by one-way ANOVA. (**E**) Neuronal cell death increased after exposure to IL-1β-treated conditioned microglial media; the effect was significantly reduced by microglia pretreatment with 50 µM berberine. (**F**) Western blot analysis showed that berberine significantly reduced the cCP-3 level compared with N2A cells treated with conditioned microglia media alone. Values are presented as mean ± SEM of four independent experiments. *P*<0.001 vs. the normal control; ^#^ *P*<0.05, ^###^ *P*<0.001 vs. N2A cells treated with conditioned microglia media alone as determined by one-way ANOVA.

Because berberine reduced the extent of neuronal degeneration after CCI, we exposed primary neurons to stretch injury to examine whether this observation was due to a direct protective effect on target neurons or was indirectly mediated by a decrease in toxic mediators. As shown in Fig. 6A, stretch injury reduced neuronal viability to approximately 60% and was not recovered with various concentrations of berberine.

Given that microglia activation and the subsequent release of various proinflammatory factors damaged neurons following TBI [33], the effects of berberine on IL-1β-induced glial activation were next determined in primary mixed glia and mouse BV2 microglial cells. In primary mixed glia, there was a low but measurable amount of NO production in the absence of IL-1β at the end of the 24 h incubation (0.79±0.08 µM). After a 24-h exposure to IL-1β, the concentration of NO in the culture media increased to 9.72±0.60 µM, but co-treatment of 5, 10, or 50 µM berberine with IL-1β for 1 day significantly reduced IL-1β-induced NO production to 44%, 42%, and 28% of that observed for the vehicle control group (all *P*<0.01; Fig. 6B). Similar results were observed in mouse BV2 microglial cells (Fig. 6C & D). The level of NO in the absence of IL-1β was low at the end of the 48h incubation (0.49±0.04 µM), and it increased to 11.30±0.73 µM after a 48-h exposure to IL-1β. Co-treatment of 1, 5, 10, or 50 µM berberine with IL-1β for 2 days significantly attenuated IL-1β-induced NO production to 65%, 20%, 17%, and 8% of that observed for the vehicle control group (all *P*<0.001).

We next assessed whether treatment of microglia with berberine conferred protection against glia-induced neuronal injury by measuring the viability of N2A cells exposed to IL-1β-treated conditioned media from BV2 microglial cells for 2 days in the presence or absence of berberine (Fig. 6C). As shown in Fig. 6E, conditioned media from 1L-1β-stimulated microglia (IL-1β–CM) significantly decreased N2A cell viability to 82.0±0.6% of the control-level (*P*<0.001). However, the viability of N2A cells stimulated with conditioned microglia media from the 50 µM berberine treatment group (IL-1β/BBR–CM) markedly increased to 93±1% (*P*<0.001; Fig. 6E). In addition, the cleaved caspase-3 level in N2A cells treated with IL-1β/BBR–CM was also significantly decreased to 18.9% of the level observed for IL-1β–CM-stimulated N2A cells (*P*<0.01; Fig. 6F). Taken together, these results indicate that the protective effects of berberine are mediated by its capacity to suppress microglial-induced injury and not through direct effects on neurons.

### Berberine inhibited IL-1β-induced activation of TLR4-mediated signaling in primary mixed glia and attenuated IL-1β-induced pro-inflammatory responses in primary microglia

**Figure 7:**
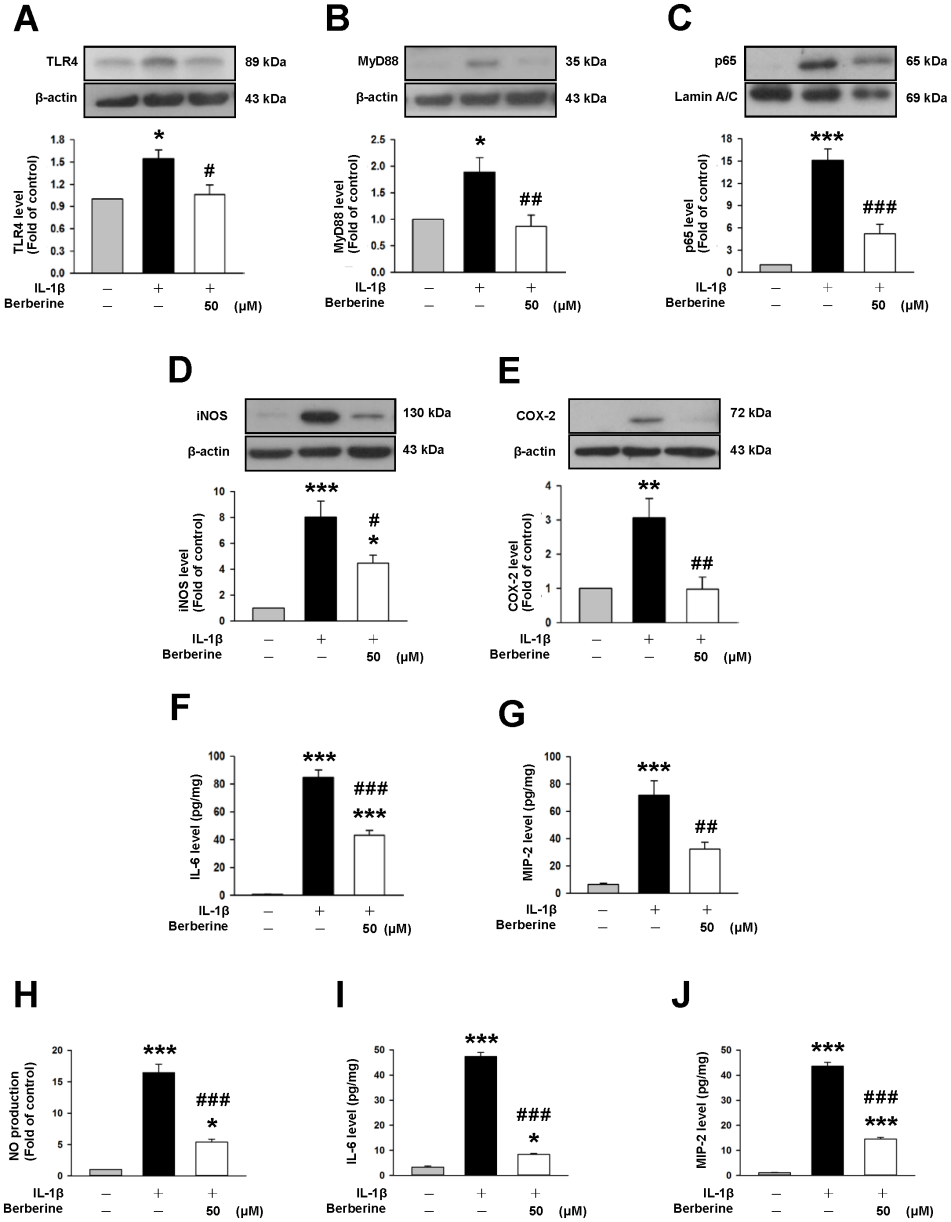
Berberine inhibited IL-1β-induced activation of TLR4/MyD88/NF-κB signaling in mixed glia and attenuated IL-1β-induced microglial activation. Representative immunoblots showing that co-treatment of 50 µM berberine with IL-1β for 1 day significantly reduced IL-1β-induced (**A**) toll-like receptor 4 (TLR4) expression (**B**) adapter protein myeloid differentiation factor 88 (MyD88) expression (**C**) p65 nuclear translocation (**D**) inducible nitric oxide synthase (iNOS) expression and (**E**) cyclooxygenase-2 (COX-2) expression by primary mixed glia. (**F, G**) Co-treatment of 50 µM berberine with IL-1β for 1 day significantly reduced IL-1β-induced secretion of IL-6 and macrophage inflammatory protein (MIP-2) as assessed by enzyme-linked immunosorbent assay from the supernatant of mixed glial cultures. Co-treatment of 50 µM berberine with IL-1β for 2 days significantly attenuated IL-1β-induced release of (**H**) nitric oxide (NO), (**I**) IL-6 and (**J**) MIP-2 from the supernatant of primary microglial cultures. Values are presented as mean ± SEM of four independent experiments. ^*^ *P*<0.05, ^**^ *P*<0.01, ^***^ *P*<0.001 vs. the normal control; ^#^ *P*<0.05, ^##^ *P*<0.01, ^###^ *P*<0.001 vs. IL-1β stimulation alone as determined by one-way ANOVA.

Previous studies have shown that TLR4/adapter protein myeloid differentiation factor 88 (MyD88)/NF-κB signaling mediates post-traumatic inflammatory responses and aggravates brain injury following TBI [19], [34]; therefore, we evaluated the effects of berberine on IL-1β**-**induced activation of TLR4-mediated pathways. In primary mixed glia, IL-1β significantly increased TLR4 and MyD88 expression as well as nuclear p65 levels (all *P*<0.05), which were significantly reduced with co-treatment of 50 µM berberine for 1 day (all *P*<0.05; Fig. 7A–C).

We next investigated whether berberine reduced the expression or production of inflammatory mediators. Treatment of mixed glia with berberine markedly suppressed the IL-1β-induced expression of iNOS to 55.8% of that observed for the IL-1β group (*P*<0.05, Fig. 7D); Following berberine treatment, COX-2 expression was reduced to 31.7% of the level in the IL-1β group (*P*<0.01, Fig. 7E). Furthermore, berberine significantly attenuated IL-1β-induced secretion of IL-6 (43.4±3.5 vs. 84.8±5.3 pg/mg, *P*<0.001, Fig. 7F) and MIP-2 (32.3±5.1 vs. 71.8±10.5 pg/mg, *P*<0.01, Fig. 7G) from the supernatant of glial cultures.

We further investigated whether berberine was capable of reducing microglia-derived inflammatory mediators using pure primary microglial cultures because activated microglia release a number of pro-inflammatory factors that contribute to secondary brain damage following TBI [33]. Co-treatment of 50 µM berberine with IL-1β for 2 days led to a significant reduction of IL-1β-induced NO production to 32.6% of that observed for the vehicle control group (*P*<0.001, Fig. 7H). Similarly, berberine significantly suppressed IL-1β-induced release of IL-6 (8.3±0.4 vs. 47.4±1.6 pg/mg, *P*<0.001, Fig. 7I) and MIP-2 (14.5±0.7 vs. 43.6±1.5 pg/mg, *P*<0.001, Fig. 7J) from the supernatant of microglial cultures.

### Delayed administration of berberine reduced neuronal death

**Figure 8:**
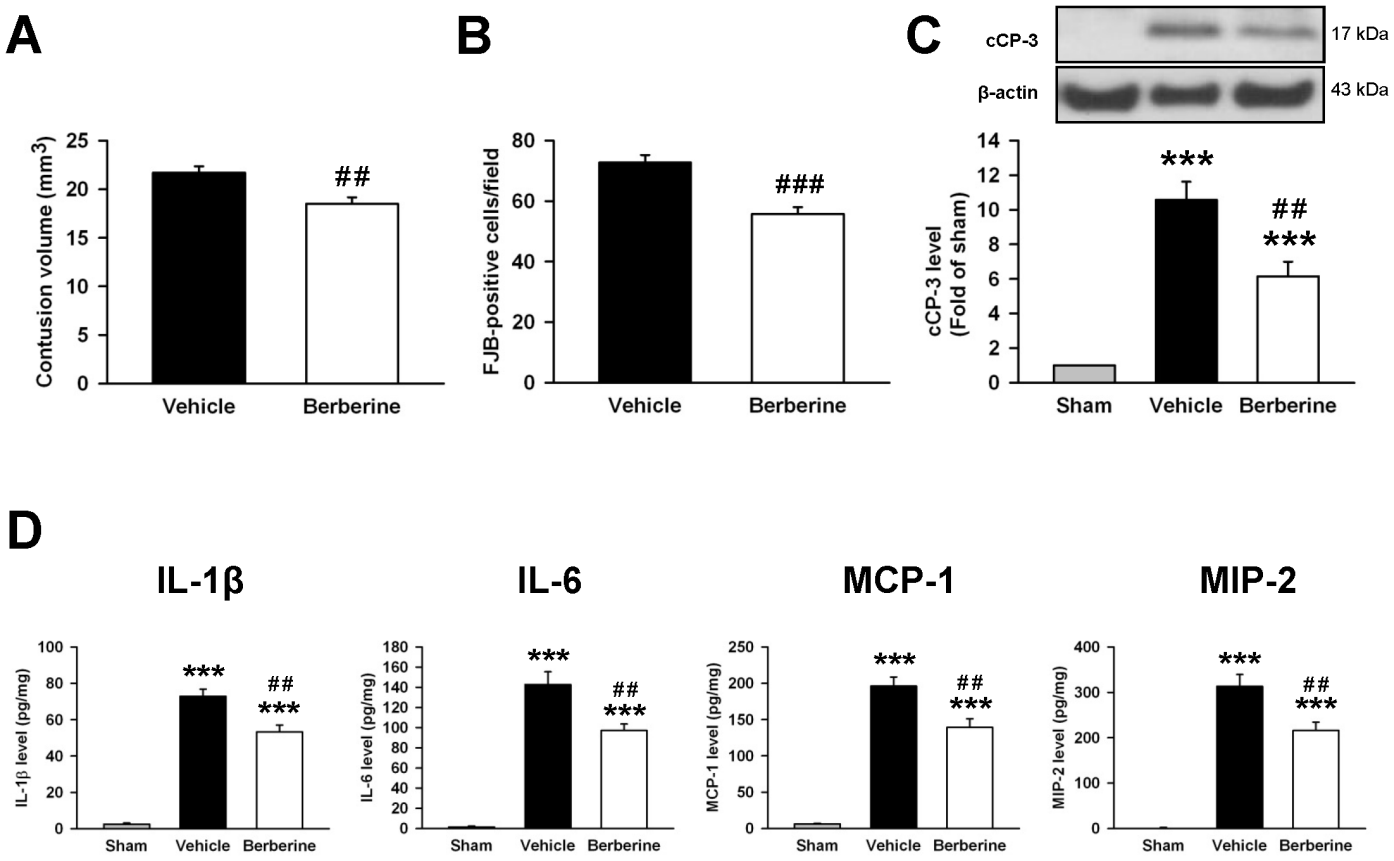
Delayed berberine treatment attenuated contusion volume, neuronal death and apoptosis and reduced inflammation after TBI. Treatment with 10 mg·kg^−1^s berberine at 3 h post-injury significantly reduced (**A**) contusion volume as assessed by cresyl violet staining, (**B**) the number of Fluoro-Jade B (FJB)-positive neurons in the cortical contusion margin and (**C**) the cleaved caspase-3 (cCP-3) level in the ipsilateral hemisphere than vehicle-treated mice at 1 day post-TBI. The total number of FJB-positive cells is expressed as the mean number per field of view (0.8 mm^2^). (**D**) Bar graphs of IL-1β, IL-6, MCP-1, and MIP-2 protein concentrations, as assessed by enzyme-linked immunosorbent assays (ELISA) in the ipsilateral cortices of sham control, vehicle-treated, and 10 mg·kg^−1^ berberine-treated mice at 1 day post-injury. Treatment with 10 mg·kg^−1^s berberine at 3 h post-injury significantly reduced IL-1β, IL-6, MCP-1, and MIP-2 protein levels compared with vehicle-treated mice. Values are presented as means ± SEM; ***P<0.001 vs. the sham control; ^#^ *P*<0.05, ^##^ *P*<0.01, ^###^ *P*<0.001 vs. the vehicle group as determined by the Student's *t*-test (n = 6 mice/group for cresyl violet and FJB stainings) or one-way ANOVA (n = 6 mice/group for Western blot analysis and n = 7 mice/group for ELISA).

To determine the therapeutic effects of delayed berberine administration following TBI, berberine treatment was delayed by 3 h post-injury. As shown in Fig. 8, berberine treatment at 3h significantly reduced contusion volume (18.5±0.7 vs. 21.7±0.6 mm^3^; *P* = 0.006; Fig. 8A) and the number of FJB-positive neurons around the injured cortical area (55.8±2.3 vs. 72.7±2.5 cells/field; *P*<0.001; Fig. 8B) compared to vehicle at day 1 post-injury. Likewise, cleaved caspase-3 was reduced by 41.8% following delayed berberine admnistration compared to the vehicle group at day 1 (*P*<0.01; Fig. 8C). Therefore, the neuroprotective effect of berberine treatment at 10 min or 3 h post-CCI was similar; contusion volume, neuronal damage and the degree of apoptosis were reduced by 14.5%, 19.4% and 45.7% when administered 10 min post-injury (Fig. 2) and 14.7%, 23.2% and 41.8% when administered at 3 h post-injury, respectively (Fig. 8).

We further determined whether delayed berberine treatment also reduced the expression of inflammatory mediators. Berberine treatment at 3h significantly reduced IL-1β, IL-6, MCP-1, and MIP-2 protein levels after CCI as compared with the vehicle group (IL-1β: 53.3±3.7 vs. 72.9±3.9 pg·mg^-1^ protein, *P*<0.01; IL-6: 97.1±6.5 vs. 142.7±12.6 pg·mg^−1^ protein, *P*<0.01; MCP-1: 139.2±12.0 vs. 196.0±12.6 pg·mg^−1^ protein, *P*<0.01; MIP-2: 215.9±18.4 vs. 313.1±26.2 pg·mg^−1^ protein, *P*<0.01, Fig. 8D). The anti-inflammatory effect of berberine treatment at 3 h is similar to that at 10 min post-injury (Fig. 5).

## Discussion

This study showed for the first time that berberine administration reduced neuronal damage and cerebral edema and improved long-term functional recovery after TBI in mice. Of particular translational significance, protection was observed even when berberine was administered 3 h post-injury. Mechanistically, berberine reduced neutrophil infiltration, microglial activation and the mRNA and protein expression of pro-inflammatory mediators; however, it did not affect Akt or Erk signaling *in vivo*. In the *in vitro* studies, we found that berberine did not directly reduce neuronal cell death in the stretch-injury model. However, berberine attenuated IL-1β-stimulated NO production in both primary mixed glia and the BV-2 cell line, which was associated with a reduced activation of TLR4/MyD88/NF-κB signaling in mixed glia. Conditioned media from IL-1β-stimulated BV-2 cells caused death in N2A cells, but treating IL-1β-stimulated BV-2 cells with berberine reduced neuronal cell death induced by microglial conditioned media. Taken together, berberine might reduce TBI-induced tissue damage by limiting the levels of inflammatory mediators produced by glial cells, rather than through a direct neuroprotective effect. Our results support the notion that inhibition of glia-mediated inflammation could protect against neuronal damage following TBI.

A single injection of berberine administered post-CCI enhanced the functional recovery in mice, which was observed as long as 1 month post-injury. Previous pharmacokinetic studies of berberine in rats revealed that it rapidly penetrates the BBB by 0.2 h after administration, peaking at 2–4 h in the brain, and was eliminated slowly [35], [36]. Thus, it is possible that a single injection of berberine conferred protection by attenuating the production of inflammatory mediators released from glial cells. Indeed, IL-β or IL-6 may affect the expression of other pro-inflammatory cytokines and chemokines during the early stages post-injury, thus amplifying the inflammatory response [37]. Our results are in agreement with previous reports in animal models of cerebral ischemia, which demonstrate that prophylactic or early berberine treatment can reduce brain tissue damage and neurological deficits at early time points (less than one week) following stroke [11]–[15]. The sustained neuroprotective effect of berberine for TBI observed in the present study is particularly important because cerebral injuries arising from different types of primary insults could cause diverse injury cascades and cellular vulnerability patterns [38]. The long-term protection is also of great clinical relevance since some agents slow down rather than prevent neuronal death [39]. Furthermore, our observation that delayed berberine administration (3 h post-injury) was sufficient to induce improvement further suggest that berberine may provide a potential therapy for TBI that is clinically feasible.

Interestingly, berberine reduced neuronal death following mouse TBI; however, it did not protect neurons in the *in vitro* stretch injury model. Although berberine also attenuated cleaved caspase 3 expression, it did not alter the phosphorylation of either Akt (both Ser 473 and Thr 308) or Erk. These results contradict previous reports from cerebral ischemia in which enhanced Akt Ser 473 phosphorylation protected neurons from apoptosis following berberine treatment [15]. It is possible that berberine can influence Akt activation that is specific for different pathological conditions. Indeed, previous studies have shown that berberine did not alter phosphorylation of Akt at either Ser473 or Thr308 in cultured endothelial cells [40]; however, it suppressed cytokine-induced activation of Akt in colon cells [41]. Also, the Akt activation reported in previous *in vitro* studies was observed in cultured neurons subjected to oxygen-glucose deprivation [12], [15] while the main insult arising from TBI in our study was mechanical stretching. An alternate explanation is that TBI may not induce the expression of heat shock protein 90 [42], which is required to maintain Akt stability under stress conditions [43].

We showed that berberine attenuated microglial activation and neutrophil infiltration, and reduced the expression of inflammatory mediators (e.g., IL-1β, IL-6, MCP-1, and MIP-2) in the injured brain. These *in vivo* findings correlated with the inhibition of IL-1β-induced upregulation of iNOS and COX2 and production of IL-6 and MIP-2 in mixed glial cultures by berberine. These findings are consistent with previous studies in which berberine inhibited amyloid-beta peptide (Aβ)- or AMP-activated protein kinase (AMPK)-induced expression of these inflammatory factors in primary microglia or microglial cell lines [7], [8]. Of the numerous neurotoxic factors released by activated glial cells, the consequences of upregulated iNOS, COX-2, cytokine and chemokine expression have been well established [44]. Also, increased levels of IL-1β, IL-6, and MCP-1 have been demonstrated in the brain or CSF of brain injured patients [45]–[47]. Along with the fact that berberine attenuated neuronal cell death induced by microglial conditioned media, these observations suggest that berberine-mediated protection of damaged neurons was mediated by inhibition of these proinflammatory factors in the injured brain.

Our results demonstrated that berberine reduced the MMP-9 activity, neutrophil infiltration and ICAM-1 expression but did not affect claudin-5 or ZO-1 expression. This reduction in inflammation was associated with a decrease in TBI-induced BBB disruption and edema formation. MMP-9 functions to degrade the extracellular matrix, including major components of the basal lamina, causing BBB disruption after TBI [27]. In addition, excessive accumulation of neutrophils causes the release of inflammatory mediators *and* reactive oxygen species, leading to BBB disruption and edema [37], [48]. Upregulation of ICAM-1 may further contribute to the intravascular collection of peripheral neutrophils. Our results suggest that the protective effect of berberine on BBB may be attributed to the reduction of MMP-9 activity, neutrophil infiltration and ICAM-1 expression in the injured brain. However, recent research found that berberine could inhibit the proliferation and activation of T cells in animal models of cerebral ischemia [11] and autoimmune neuritis [49]. Also, berberine decreased lipopolysaccharide-induced proinflammatory responses in cultured murine neutrophils via activating AMPK [50]. TBI causes a systemic inflammatory response including increased plasma levels of inflammatory mediators and circulating neutrophil counts [48], both of which contribute to TBI-induced brain damage [37]. Therefore, it would be important in future studies to explore the peripheral effect by which berberine protects against TBI.

We further investigated the effects of berberine on the TLR4/MyD88/NF-κB signaling pathway, which participates in the cerebral inflammation in TBI [19], [34]. The TLR4 signaling pathway is mediated by MyD88, which activates NF-κB, resulting in the upregulation of many pro-inflammatory genes (e.g., cytokines, chemokines, COX-2, and iNOS) [51]. These proinflammatory mediators can then further activate NF-κB, forming a positive feedback loop to amplify inflammatory signals. In the present study, berberine attenuated TLR4 and MyD88 protein expression as well as nuclear NF-κB; it also abrogated the CCI-induced increase in inflammatory mediators, including COX-2, iNOS, IL-6, and MIP-2. Our results are in accordance with a recent study demonstrating that berberine reduced lipopolysaccharide-induced intestinal injury by suppressing the activation of TLR4 and NF-κB in the ileum [10]. However, our data do not clarify the mechanism by which inhibiting glial activation attenuated neuronal apoptosis. One possibility is that berberine may suppress extrinsic apoptosis via inhibiting TLR4 signaling as MyD88 can bind Fas-associated death domain protein (FADD) to trigger apoptosis via caspase-8 activation [52]. Further investigations are needed to clarify the mechanisms by which berberine modulates inflammation to influence neuronal apoptosis.

W chose the route of intraperitoneal administration based on previous two neuroprotective studies in experimenal cerebral ischemia which showed that intraperitoneal injection of berberine (5 mg/kg for 2 doses, 0.5 h and 13.5 h after surgery) [11] and 10 mg/kg, immediately after surgery) [14] protected against ischemic brain injury. To our knowledge, there have been no pharmacological studies directly investigating brain distribution of berberine following intraperitoneal injection. However, the brain level of berberine increased at about 0.2 h after intravenous administration and peaked at 2–4 h [35], [36]. Although we didn't demonstrate that berberine could penetrate through the BBB after systemic administration, previous studies, both by others and ourselves [19], [53], [54], have shown that cortical impact injury induced BBB disruption starting at 5min post-injury and continued through 7 days. This opening of the BBB helps the transfer of systemically-administered drugs into brain cells. A recent study further demonstrated that berberine remained relatively stable in the brain after oral administration [55]. Further investigations will be required to establish the route of administration to facilitate clinical applications of berberine to human TBI patients.

In conclusion, our results show that post-injury administration of berberine protects against brain damage in a clinically relevant model of TBI. This neuroprotective effect is at least in part mediated by inhibition of the TLR4/MyD88/NF-κB signaling pathway in glial cells. Our findings emphasize the role of glial cells in the pathogenesis of TBI; by suppressing their proinflammatory response with anti-inflammatory agents, we may inhibit the neurotoxicity associated with this disease. Considering the extended therapeutic window of berberine and its long-lasting effects, together with the fact that it has been extensively used preclinically and clinically, our results suggest that berberine could be a potential therapeutic agent for TBI.

## Supporting Information

S1 Fig **Effects of 3 different doses of berberine in contusion-injured mice.** (**A**) Treatment with 5 mg·kg^−1^ berberine did not significantly alter rotarod performance compared with the vehicle-treated group. Mice treated with 10 mg·kg^−1^ and 15 mg·kg^−1^ berberine had better rotarod performance than vehicle-treated mice at 1, 4 and 7 days post-CCI. (**B**) There was no significant difference between the 5 mg·kg^−1^ berberine-treated and vehicle-treated groups at all tested time points in the beam walk test. Beam walk latencies were significantly shorter for both the 10 mg·kg^−1^ and 15 mg·kg^−1^ groups than the vehicle group at 4 and 7 days post-CCI. (**C**) The mNSSs were significantly lower in the 10 mg·kg^−1^ berberine group than the vehicle group at all tested-time points and lower in the 15 mg·kg^−1^ berberine group than the vehicle group at 1 and 7 days. Values are presented as mean ± SEM; ^#^ *P*<0.05, ^##^ *P*<0.01, and ^###^ *P*<0.001 vs. the vehicle control group as determined by two-way ANOVA. (n = 12 mice/group).
(TIFF)
