# Supplementary figures and images for: Berberine Protects against Neuronal Damage via Suppression of Glia-Mediated Inflammation in Traumatic Brain Injury
Source: PLoS One. 2014 Dec 29;9(12):e115694. doi: 10.1371/journal.pone.0115694 (PMC4278716; doi:10.1371/journal.pone.0115694)

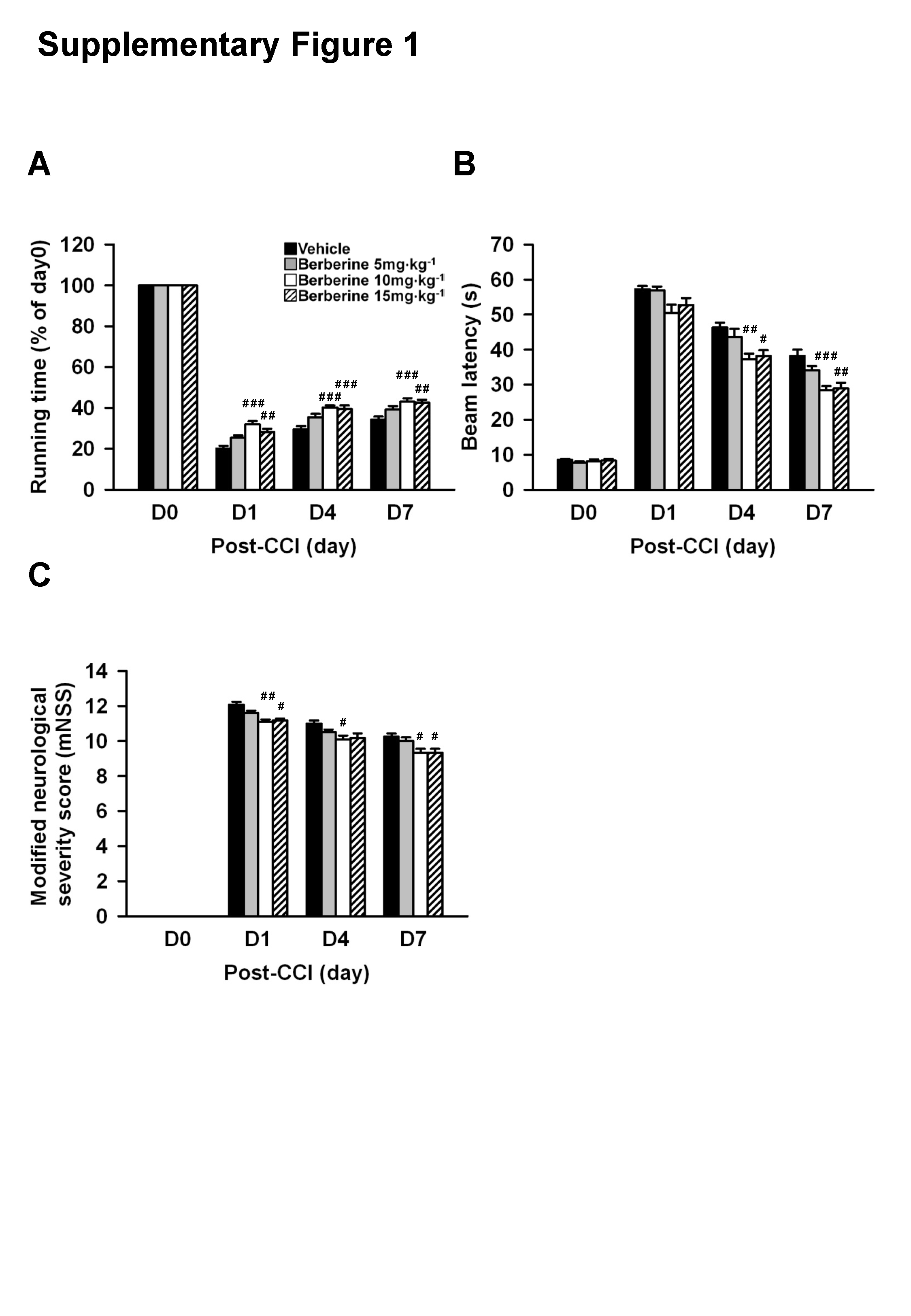

Supplement: S1 Fig — Effects of 3 different doses of berberine in contusion-injured mice. (A) Treatment with 5 mg·kg−1 berberine did not significantly alter rotarod performance compared with the vehicle-treated group. Mice treated with 10 mg·kg−1 and 15 mg·kg−1 berberine had better rotarod performance than vehicle-treated mice at 1, 4 and 7 days post-CCI. (B) There was no significant difference between the 5 mg·kg−1 berberine-treated and vehicle-treated groups at all tested time points in the beam walk test. Beam walk latencies were significantly shorter for both the 10 mg·kg−1 and 15 mg·kg−1 groups than the vehicle group at 4 and 7 days post-CCI. (C) The mNSSs were significantly lower in the 10 mg·kg−1 berberine group than the vehicle group at all tested-time points and lower in the 15 mg·kg−1 berberine group than the vehicle group at 1 and 7 days. Values are presented as mean ± SEM; # P<0.05, ## P<0.01, and ### P<0.001 vs. the vehicle control group as determined by two-way ANOVA. (n = 12 mice/group). (TIFF) [file pone.0115694.s001.tiff]
